# Qualitative and quantitative proteomic analysis of Vitamin C induced changes in *Mycobacterium smegmatis*

**DOI:** 10.3389/fmicb.2015.00451

**Published:** 2015-05-18

**Authors:** Abhishek Mishra, Dhiman Sarkar

**Affiliations:** Council of Scientific and Industrial Research-National Chemical Laboratory, Organic Chemical Division, Combichem Bioresource CenterPune, India

**Keywords:** vitamin C, *Mycobacterium smegmatis*, dormancy, proteomics, tuberculosis

## Abstract

Vitamin C is a critical dietary nutrient in human which has a wide range of regulatory effects on gene expression and physiology of *Mycobacterium tuberculosis* that leads to a dormant drug-tolerant phenotype. In the presence of iron, vitamin C shows a high bactericidal activity even in the drug resistant phenotype of *M. tuberculosis*. The regulatory mechanisms underlying vitamin C induced adaptations are largely unknown due to lack of functional genomics data in this field. In this study, we attempt to characterize the direct effect of vitamin C treatment on the physiology of actively growing *Mycobacterium smegmatis*. The study chose *M. smegmatis* as it is a fast-growing bacterium and a non-pathogenic model system which shares many physiological features with the pathogenic *M. tuberculosis* including dormancy and its regulation. The proteomic adaptation of *M. smegmatis* on vitamin C treatment demonstrates the important changes in cellular and metabolic process such as reversal of tricarboxylic acid cycle, decrease in ATP synthesis, decrease in iron acquisition and storage, and induction of dormancy regulators WhiB3, PhoP, and Lsr2.

## Introduction

Tuberculosis (TB) which is only second to HIV/AIDS, is one of the deadliest infectious diseases of our time, causing 1.3 million deaths in 2012 (WHO, 2014). The World Health Organization (WHO) reports suggest that one third of the world's population is infected with latent TB. The etiologic agent, *Mycobacterium tuberculosis*, has the ability to survive in the latent stage for years before reactivation and subsequent induction of full-fledged disease. The cornerstone of TB control efforts has been compromised by the outbreak of MDR (multi drug resistance) and XDR (extremely drug resistance) *M. tuberculosis*. In 2012 WHO estimated about 450,000 people who developed the multidrug-resistant TB (MDR-TB) and there were about 170,000 deaths from MDR-TB across the globe. The first-line drugs isoniazide and rifampicin potentially kills 99.0–99.9% of *M. tuberculosis* cells *in vitro* within 4–7 days. However, resistance to these bactericidal drugs develops very fast under both *in vitro* and *in vivo* conditions. Therefore, there is an urgent need to discover potential anti-microbial compounds as well as develop new methods for the prevention and treatment of disease, more importantly, new therapies that provide longer sustainability and effectiveness against bacterial pathogens.

In humans, vitamin C is an essential dietary micronutrient with antioxidant property which is involved in a wide range of vital cellular and physiological functions (Mandl et al., 2009). Vitamin C not only affects a number of metabolic reactions and biological processes but also have medical relevance in curing various diseases. The vitamin C supplement in human diet has led to the elimination of scurvy (Hodges et al., 1969) and has proven to be beneficial in the treatment of viral and bacterial infections (McCormick, 1951; Pauling, 1976; Padayatty et al., 2003). In 1976, Linus Pauling advocated a high dosage (1–3 g/day) of vitamin C for the prevention of common cold and flu. Vitamin C also demonstrated bacteriostatic and bactericidal effect in several pathogenic bacteria like *Staphylococcus aureus*, *Escherichia coli*, and *Streptococcus* sp. A number of reports have described an improved effect of orally administered vitamin C in preventing and treating TB infection in human and guinea pig (McConkey and Smith, 1933; Hemila et al., 1999), while other studies have reported the deficiency of vitamin C in TB patients (Andosca and Foley, 1948). The action of vitamin C in infectious disease is generally attributed to the effects on host cell mediated immunity, such as enhanced T- cell response and migration of leukocytes to the site of infection (Field et al., 2002). Furthermore, it protects host from oxidative and nitrostative damage occurring due to generation of reactive oxygen species and reactive nitrogen species to kill intracellular pathogen (Jariwalla and Harakech, 1996). Pichat and Reveilleau postulated that the bactericidal activity of vitamin C could be due to vitamin C itself or its decomposition product(s) (Pichat and Reveilleau, 1950, 1951). As a defense mechanism the host immune cells have the ability to concentrate ascorbic acid to millimolar levels intracellularly (Bergsten et al., 1990; Washko et al., 1993; Welch et al., 1995; Laggner et al., 1999).

In a recent report, vitamin C induced a dormancy phenotype, which showed hallmark features like bacteriostasis and drug tolerance, in aerobically growing *M. tuberculosis* (Taneja et al., 2010). Similarly, another study demonstrated a correlation between the high vitamin C content of some medicinal plant extracts and their potent activity against *M. tuberculosis* (Narwadiya et al., 2011). Furthermore, vitamin C enhances the bactericidal activity against *M. tuberculosis* in the presence of a transition metal like ferrous that leads to the generation of reactive oxygen species by Fenton's reaction and affects several biological processes (Vilchèze et al., 2013). These studies suggest that vitamin C directly affect pathogen physiology. Vitamin C has both anti-oxidant and a pro-oxidant property and hence the sterilizing effect of vitamin C on *M. tuberculosis* cultures remained controversial (Taneja et al., 2010; Vilchèze et al., 2013).

An incomplete understanding of the physiology of bacteria associated with vitamin C has been an impediment toward the therapeutic advancement of vitamin C. In this report we attempt to characterize the effect of vitamin C treatment on the proteome profile of *Mycobacterium smegmatis*, a non-pathogenic organism closely related to *M. tuberculosis*. *M. smegmatis* share hallmark features of dormancy with *M. tuberculosis*, making it a suitable model system for this study. The availability of the complete genome sequences for *Mycobacterium* strains have made bioinformatics, genomics, and proteomics studies more precise to obtain a better understanding of the pathophysiology of *Mycobacterium* sp. Currently, it is easier to highlight pathways with higher sensitivity, to explore a rational drug-discovery program (Smith et al., 1997; Cole et al., 1998; Garnier et al., 2003). In comparison to mRNA studies, proteomic analysis is more focused on functional products, consequently, providing a more accurate assessment of the conditional changes. Traditionally, two-dimensional gel electrophoresis coupled with mass spectrometry (2D-PAGE-MS) is used for protein identification and quantification to study conditional changes in the sample. In contrast to its popularity, this method remains ambiguous due to its low load ability which results in multiple spots for a single protein or multiple proteins in a single spot. Along with poor separation of hydrophobic, acidic or alkaline proteins, quantitative analysis of proteins remained uncertain due to undetermined post-translational modifications, protein degradation, protein isoforms and variability in tryptic digest recovery from the gel. To compensate for these limitations of 2D-PAGE-MS, an advanced gel-free and label free method had been developed (Silva et al., 2005). We used the same method with some modifications for proteomic characterization of vitamin C treated *M. smegmatis* cells using Liquid Chromatography- elevated energy Mass spectrometry (LC/MS^E^).

## Materials and methods

### Chemicals, culture maintenance, and media

All the chemicals used in the study were purchased from Sigma-Aldrich, USA, except Dubos medium which was purchased from DIFCO, USA. The stock culture of *M. smegmatis* strain ATCC 607 was maintained at −70°C on Dubos-agar slants and subcultured once in Dubos medium before inoculation to defined experimental media.

### Dose response of *M. smegmatis* in the presence of ascorbic acid

The inhibitory activity of vitamin C on growing *M. smegmatis* was tested by following the method previously described (Khan and Sarkar, 2006). Briefly, 1% mid-log phase of a culture at a cell density of 1 O.D._620nm_ was inoculated in 20 ml of defined medium (0.5 g KH_2_PO_4_, 0.2 g sodium citrate, 60 mg MgSO_4_, 0.5 g asparagine and 2 mL glycerol in 100 mL of distilled water at pH 6.6) in a 100 ml conical flask and was incubated for 3 days under aerobic conditions at 37°C with shaking at 150 rpm (Thermo Electron Model No. 481; Thermo Electron Corp., Marietta, OH). Vitamin C was added into the defined medium at the time of inoculation at the final concentration ranging from 0.125 to 2.0 mM, with negative control being an equal volume of only defined medium. Growth of *M. smegmatis* was estimated after a 3 day incubation period by determining the colony forming units (CFU/ml).

### Preparation of *Mycobacterium smegmatis* whole cell proteome

For comparative quantitative analysis of proteins in active bacilli and vitamin-induced bacilli, 0.1% mid-log phase of a culture at a cell density of 1 O.D._620nm_ was inoculated into 200 ml of defined medium in a 1000 ml conical flask and was incubated under aerobic conditions at 37°C with shaking at 150 rpm. Once culture reached 0.5 O.D._620nm_, 2 mM of Vitamin C at final concentration was added in defined medium and for control an equal volume of only defined medium was added and was incubated under aerobic conditions at 37°C with shaking at 150 rpm. After 6 h, 250 ml 0.5 O.D_600_ of the bacterial cells were harvested by centrifugation at 10,700 g for 30 min. The pellet was resuspended in extraction buffer (urea 8 M, thiourea 2 M, dithiothreitol 40 mg/ml and 3-[(3-Cholamidopropyl)dimethylammonio]-1-propanesulfonate (CHAPS) 2%). After resolubilization, sonication was done at 75 Hz with 30 s pulse with a break of 45 s. This cycle was repeated for eight times. Proteins were precipitated by trichloroacetic acid and Acetone extraction in the ratio of 1: 8 to the protein sample. This consortium was kept at −20°C for 2 h and then centrifugation was done at 16,800 g for 1 h. The precipitate was further solubilized in extraction buffer and used for further studies. In each sample, concentrations of proteins solubilized in extraction buffer were estimated using the One Plus 2D quantitation kit (Amersham Biosciences, USA).

### Trypsin digestion of *Mycobacterium smegmatis* whole proteome

Approximately, 10 μg complex protein mixture was taken and washed twice with 50 μL of 0.1% Rapigest™ (Waters Corporation, Milford, MA) (1 vial diluted in 1000 μL 50 mM ammonium bicarbonate) using a MW 5000 (or MW 3000) cut off spin column. Protein solution was concentrated to 10–20 μL using a MW 5000 (or MW 3000) cut-off spin column. The concentrated protein solution was then heated to 80°C for 15 mins and then 3 μL of 100 mM dithiothreitol (prepared in 50 mM ammonium bicarbonate) was added before heating at 60°C for 15 min. Then 3 μL of 200 mM Iodoacetamide (made up in 50 mM ammonium bicarbonate) was added and the solution was left in the dark at room temperature for 30 min. 1 μL of 1 μg/μl (2 μL of 0.5 μg/μl) trypsin (prepared in 50 mM ammonium bicarbonate) was added and the solution was left overnight at 37°C. 1 μL of concentrated HCl was added and the solution was incubated at 37°C for 20 min before mixing and later centrifuged at 36,000 g for 30 min. The supernatant was transferred to a clean eppendorf tube and the digest was transferred to nano LC/MS^E^ analysis.

### LC/MS^E^ analysis

For LC/ MS^E^ an earlier method was used with modifications (Silva et al., 2005). Briefly, each trypsin digested protein sample was spiked with an internal standard (tryptic digest of alcohol dehydrogenase from *Sacchromyces cervesie*) at a level of 50 fmol per 10 μ L injection before LC/MS^E^ analysis. For sample analysis, 10 μ L aliquots of proteome tryptic digests were analyzed in triplicates by LC/MS^E^ using a nano ACQUITY ultra pressure liquid chromatograph and Premier Quadrupole-Time of Flight (Q-ToF) mass spectrometer equipped with a nanolock spray ion source (Waters Corporation, USA). Samples, at a flow rate of 10 μ L/min, were injected onto a C18 trapping cartridge (300 μm i.d. × 1 cm length, Waters Corporation, USA). Peptides were further separated, at a flow rate of 300 nL/min using a linear gradient from 2 to 40% B over 120 mins (A = 0.1% formic acid in water, B = 0.1% formic acid in acetonitrile), by using gradient elution onto a 75 μm i.d. × 25 cm column, 1.7 μm particle size, packed with BEH C18 Stationary phase (Waters Corporation, USA). Precursor ions and associated fragments were acquired by operating the Q-Tof in the LC/MS^E^ mode of acquisition, where alternating 2 s scans of low (4 V) collision energies were used to generate either intact peptide ions or high (10–32 V) collision energies were used to generate peptide product ions. An external standard for mass calibration, Glu-fibrino peptide at a concentration of 200 fmol/μ L (*m*/*z* 785.8426) was injected through the nanolock spray ion source at a constant flow rate of 600 nL/min and monitored every 30 sec. Samples were injected as sets based on the treatment from earliest to latest time points. The sensitivity and performance of the instrument was monitored by analyzing a protein standard (tryptic digest of bovine serum albumin) prior to the first sample injection and again following the last sample injection.

### Data processing and database searching

Protein Lynx Global Server V 2.2.5 software (PLGS 2.2.5) (Waters Corporation, USA) was used to process acquired raw data files to generate precursor mass lists as well as associated product ion mass lists for subsequent protein identification and quantification. After processing each data file was searched against the Uniprot protein database (http://www.uniprot.org/) using the IDENTITY^E^ database search algorithm within PLGS 2.2.5. Before searching processed data files, the internal standard alcohol dehydrogenase from *Sacchromyces cerevisiae* sequence was added to the database to enable the absolute protein quantification functionality in each sample. The precursor intensity measurements acquired from the output file was used to determine the relative quantification of protein. For each protein, the redundant quantitative measurement obtained from the multiple tryptic peptides were used to determine an average relative fold change. For each average fold-change a 95% confidence interval was determined from the standard deviation of total number of tryptic peptides. For mass accuracy (10 ppm for precursor ions and 15 ppm for product ions) a default search parameters was used with an “automatic” setting at minimum for 1 peptide match per protein, 3 consecutive product ion matches per protein, and 7 total product ion matches per protein. During the database search a maximum of 1 missed tryptic cleavage site was allowed.

## Results

### Effect of vitamin C on growth of *M. smegmatis*

**Figure 1:**
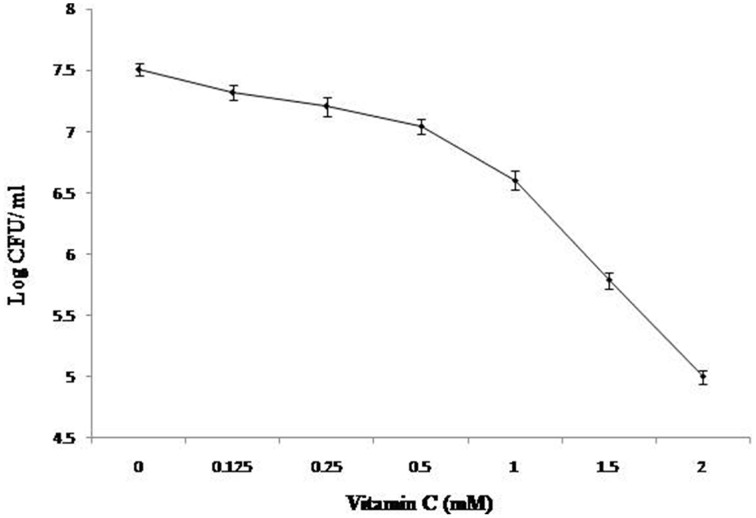
**Effect of Vitamin C on the growth of bacilli**. 0.1% of ~1.0 OD_620_ *M. smegmatis* culture was inoculated in defined medium containing different concentration of Vitamin C and was kept at 37°C at 150 rpm under identical conditions. After 3 days, CFU counts were taken as described in “Materials and Methods.”

In this study, we used defined medium to emphasize the comparable growth of the organism/s with respect to changes in composition of media. Also, it avoids the interference due to commonly used complex medium for *Mycobacterium* sp. growth. Earlier it has been shown that under aerobic condition and Wayne 0.5 headspace ratio model the growth of *M. smegmatis* was comparable in both defined and other complex media (Khan and Sarkar, 2006). Earlier reports suggest that high concentrations of vitamin C inhibit the growth of various bacterial species, some of which include *Helicobacter pyroli*, *Staphylococcus aureus*, and *M. tuberculosis* (Zhang et al., 1997; Taneja et al., 2010; Narwadiya et al., 2011; Kallio et al., 2012; Vilchèze et al., 2013). In order to validate same in *M. smegmatis*, vitamin C was tested in aerobic condition at different concentration. The effect on growth was observed by adding vitamin C at different concentrations in the culture at the time of inoculation and experiment was terminated at 3 days of incubation (as described in “Materials and Methods”). It was observed that cell number was reduced by 1.7 and 2.5 log when vitamin C was used at 1.5 and 2.0 mM concentration, respectively (Figure 1). At 2 mM concentration of vitamin C more than 90% of inoculated cells achieved bacteriostasis. Therefore, 2 mM concentration was used for the proteomic characterization study.

### Global expression change in vitamin C treated and active bacilli

**Figure 2:**
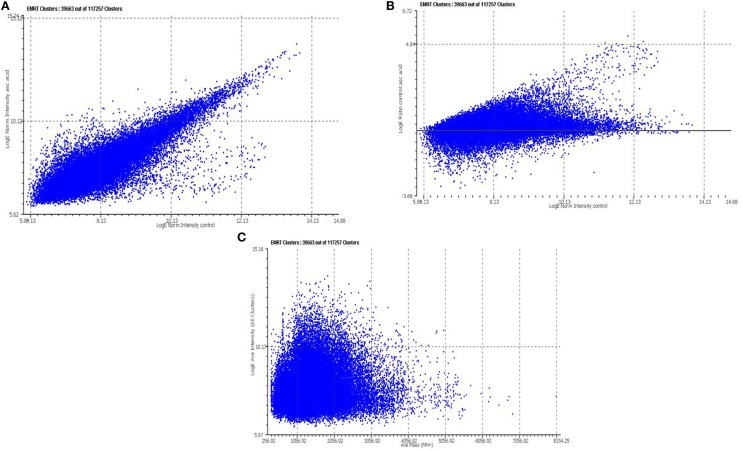
**Relative quantitative analysis of tryptic peptide identified by LC/MS^**E**^**. Each peptide component detected by LC/MS^E^ is annotated as an EMRT (*exact*-*m*ass, *r*etention *t*ime pair) **(A)** Binary comparisons of the log intensity measurements obtained from the EMRTs for Vitamin C treatment vs. Control **(B)** The natural log of the intensities (Vitamin C treated vs. control) plotted against the natural log of the replicating ions detected in the control sample. **(C)** The natural log of the average intensities (all clusters) plotted against average Mass. For each protein, the redundant quantitative measurement obtained from the multiple tryptic peptides were used to determine an average relative fold change. For each average fold-change a 95% confidence interval was determined from the standard deviation of total number of tryptic peptides.

Tryptic peptides generated for 2 mM vitamin C treated and control samples were analyzed by LC/MS^E^ in triplicate. The complete list of detected peptides was converted to a text file containing all of the mass spectrometric and retention time data using MASSLYNX and PLGS 2.2.5 software (Waters Corporation, USA), for data quality assessment. Each detected peptide component is annotated as an EMRT (*exact*-*m*ass, *r*etention *t*ime pair). Quantitative analysis was performed using the triplicate of same set of experiments. Figure 2A illustrates the binary comparison of replicate injections for the average intensity measurements between control and vitamin C treated cells. The average standard deviation was ~0.32 between the relative intensity ratio of matched peptide components of the control and 2 mM vitamin C treated cells. The natural logarithm of the ratio of the average intensities from both the condition, vitamin C treated (denominator) vs. control (numerator), was plotted against the average intensity of the total number of matched peptide components between the vitamin C treated and control samples, Figure 2B. Similarly, the natural log of the average intensities (all clusters) plotted against average Mass, Figure 2C. The observed relative fold change from the matched peptide components of the samples, control and vitamin treated, were plotted to identify peptide components of interest (Figure 2B). This method provides a useful filter which allows one to perform a search with a decreased but robust subset of peptide components. Total 463 peptide components were identified which exhibited a log intensity fold change range from 0.19 to 6.4.

About 818 proteins with pI ranging from 3 to 11, were identified using multiplex LC-MS^E^ technique in *M. smegmatis* grown in aerobic and vitamin C treated cultures. By using PLGS V 2.2.5 analysis software result of three independent experiments identified 185 proteins against *M. smegmatis* database with significant folds of change in expression of proteins (The complete list of these proteins is provided as complementary data).

### Energy and fatty acid metabolism

Like other bacterial system, glucose in mycobacterium is largely oxidized through glycolysis and to some extent through the pentose phosphate pathway (PPP) (Jayanthi et al., 1975). Glycolysis yields metabolites of central metabolism such as pyruvate, phosphoenol pyruvate and acetyl-CoA, whereas, PPP provides pentose sugar (ribose 5-P for nucleotide synthesis) and reducing equivalents in form of NADPH for reductive biosynthetic reactions. A derivative of fatty acids or sugars catabolism, Acetyl CoA, is assimilated through the Krebs cycle or tricarboxylic acid (TCA) cycle. TCA cycle provides biosynthetic precursors and reducing equivalents for energy generation and biosynthetic reactions. Enumeration of proteins encoding key glycolytic, TCA cycle and PPP enzymes of *M. tuberculosis* were down-regulated upon vitamin C treatment. However, succinate dehydrogenase differs in response and was up-regulated in vitamin C treated cells. This was in line with previous reports where succinate dehydrogenase was up-regulated in response to Wayne hypoxia and mouse *in vivo* model and this upregulation was found to be associated with a remarkable accumulation of succinate in the extracellular environment (Shi et al., 2010; Watanabe et al., 2011). By analyzing radiolabeled isotopomers of succinate authors concluded that this succinate is an outcome of the TCA cycle switch toward the non-oxidative direction in oxygen limiting condition (Watanabe et al., 2011).

Similarly, proteins encoding important genes of fatty acid metabolism FadA and FadD and mycolic acid synthesis, FabG, are down-regulated in vitamin C treated cells. However, Cyclopropane fatty acyl phospholipid synthase was uniquely expressed in vitamin C treated cells. Cyclopropanation of mycolic acids is uncommon in the cell wall of saprophytic species such as *M. smegmatis*, however, this modification is associated with pathogenic bacteria like *M. tuberculosis*, *M. bovis*, and *M. Chelonae* (Minnikin et al., 1982). In addition, cyclopropane ring modifications are found to have subtle effects on the fluidity and permeability of the cell wall which compromises the ability of bacteria to resist to oxidative stress (George et al., 1995; Yuan et al., 1995). Similarly, in well studied *E. coli* and other bacterial species that possess ability to cyclopropanate their plasma membrane, this phenomenon occurs during the transition from active growth to stationary phase, and as a response to unfavorable environmental conditions such as low pH, high temperatures and low oxygen availability (Wang and Cronan, 1994; Grogan and Cronan, 1997).

### ATP related

ATP synthase, an essential enzyme in the energy metabolism, is a validated drug target for the treatment of TB, and ATP synthase inhibitors, such as diarylquinoline, is a promising drug candidate which is potent against both active and dormant forms of *M. tuberculosis* (Welch et al., 1995). ATP synthase is a multi-subunit complex consists of a membrane-embedded F_0_ part and a cytosolic F_1_ moiety (Welch et al., 1995). We found down-regulation of major components of ATP synthase, ATP-αβ γδ ε, by 2.6–3 folds in comparison to vitamin C treatment (**Table 2** and supporting online material). This enzyme is involved in ATP synthesis by utilizing the proton-motive force (PMF) across the bacterial cytoplasmic membrane. In several bacteria this reaction can be reversed and ATP hydrolysis is utilized to maintain PMF, this strategy assist bacteria to survive in the low oxygen concentration environments (Andries et al., 2005). Previous report suggests a strong down- regulation of ATP synthase is characteristic of dormant bacilli in different models (Boyer, 2002).

### Transcription and translation associated

The HspX protein is a dominant antigen produced by *M. tuberculosis* during the latent stage of infection in different animal model systems as well as in models that aim to mimic granulomatous environment *in vitro*. Along with its association with latency, it is also expressed during static growth, upon entry into macrophages or under oxygen deprived condition (Yuan et al., 1996, 1998). The earlier reports proposed the role of HspX in enhancing the stability of proteins and cell structures, which consequentially helps the bacilli maintain long-term survival in adverse condition (Yuan et al., 1996; Cunningham and Spreadbury, 1998). Interestingly, HspX induces both B-cell and T-cell responses in patients with active TB as well as in healthy people with latent infection (Davidow et al., 2005; Demissie et al., 2006). In our study, HspX (MSMEG_3932) expressed exclusively in vitamin C treated cells and remained undetected in control samples.

PhoP and Lsr2 are two important transcriptional regulators that are uniquely expressed in vitamin C treated cells (Table 1). Lsr2, is a *M. tuberculosis* analog of the H-NS nucleoid binding protein (Colangeli et al., 2009; Gordon et al., 2010), whereas, PhoP mediates a range of adaptation to enduring hypoxia response, including upregulating DosR (Gonzalo-Asensio et al., 2006, 2008; Ryndak et al., 2008). These claims are strengthened by proposed regulatory network based on CHIP-sequencing as PhoP binding to DosR is the strongest among 50 transcription factors (Galagan et al., 2013). Furthermore, it also confirmed direct binding between PhoP and the aprABC locus required to mediate pH adaptation (Abramovitch et al., 2011; Galagan et al., 2013). PhoP and Lsr2 bind upstream and directly regulate WhiB, an essential gene (Gomez and Bishai, 2000), which is more than 20-fold induced at late stationary phase, and more than 10-fold induced at low pH (Geiman et al., 2006). In addition to unique expression of PhoP and Lsr2, WhiB a dormancy regulator is up-regulated by more than 3-folds in vitamin C treated mycobacterial cells (Table 1). The WhiB-like proteins are unique to *Actinomycetes*, was first reported in *Streptomyces coelicolor* and found to be essential for sporulation (Davis and Chater, 1992; Soliveri et al., 2000). They are small proteins (75–130 amino acids) with a conserved aspartate and a helix-turn-helix-like motif and 4 conserved cysteine residues quintessential for metal-coordinated DNA-binding proteins (Smith et al., 1997). These 4 cysteines bind to a [4Fe-4S] cluster which changes the conformation of the protein in a redox-dependent manner (Jakimowicz et al., 2005; Singh et al., 2007). The *M. tuberculosis whiB* genes are induced independently by various stimuli like gradual oxygen depletion of the Wayne hypoxia model, and *in vivo* mouse model, cyclic AMP and nitric oxide addition, may add versatility to their suggested redox-sensing properties (Larsson et al., 2012).

**Table 1:** **Summary of proteins up-regulated in Vitamin C treated cells**.

**Uniprot accession no**.	**Protein description**	**PLGS score**	Control: Vitamin C Log(e)Ratio^*^
A0QT07	Succinate dehydrogenase	1130.6	−1.14 ± 0.29
A0R5R5	Cyclopropane fatty acyl synthase	602.28	Unique^**^
A0QZ83	14 kDa antigen hspX	3274.64	Unique^**^
A0R4L1	PhoP	876.78	Unique^**^
A0R576	Lsr2	1230.32	Unique^**^
A0QWV9	WhiB	1367.16	−0.99 ± 0.22
A0R5R1	Nucleoid associated protein	26950.02	−1.35 ± 0.35
A0R4H0	29 kDa antigen	1500.41	Unique^**^
A0QW82	Uncharacterized protein	1571.4	Unique^**^
A0R2E3	Uncharacterized protein	7595.02	−1.12 ± 0.15
A0R1B5	Uncharacterized protein	3607.62	−1.05 ± 0.15
A0R2J4	Uncharacterized protein	2426.8	−0.96 ± 0.2

* The natural log of the average relative intensity measurement for associated peptide ions from control divided by the average relative intensity measurement for the same ions found in the Vitamin C treatment.

** Unique indicates a protein not detected in control.

Bacterial chromosomes are primarily packaged into sub-micrometer sized compact structures, termed as nucleoids. The structure and dynamics of nucleoids is determined by the combination of several factors like DNA supercoiling, macromolecular crowding and architectural nucleoid-associated proteins (NAP). The emerging model of suggest that NAP not only associated with the structure of the chromosomes but are also involved in process like replication, recombination, repair and transcription (Dillon and Dorman, 2010). NAP is identified as an essential gene for *M. tuberculosis* in high-density mutagenesis and deep sequencing exploration studies (Sassetti et al., 2003; Sassetti and Rubin, 2007; Griffin et al., 2011). Furthermore, the small molecule inhibitors of NAP- HU disrupted nucleoid architecture and reduced *M. tuberculosis* growth (Bhowmick et al., 2014). We found that NAP (MSMEG_6280) is up-regulated by 4-folds in *M. smegmatis* upon vitamin C treatment. The strong up-regulation of NAP suggests that it has an important role in transcription regulation during adaptation to vitamin C treatment. Increase expression of NAP may have pre-emptive role in silencing energy metabolism genes and preserving nucleoid structure.

Prokaryotic ubiquitin like Protein, Pup, is down-regulated by 3-fold in vitamin C treated cells which is known to target proteins for degradation by mycobacterium proteosome. Pupylation is an interesting protein interaction phenomenon and only known post-translational modification system in prokaryotes but the understanding of this process and its implication is still at infancy.

### Cell wall and cell division

An optimal level of *M. smegmatis* FtsZ was required to sustain cell division and that the cell division initiation mechanism was also similar in other mycobacterial species (Dziadek et al., 2003). Ftsz is a structural homolog of tubulin function as cell division initiator in a GTP- dependent process. Its controlled interaction with other cell division proteins in spatial and temporal manner is considered as key event to bacterial cell division (Dziadek et al., 2003). It was found that expression of FtsZ was decreased by 2.6-fold upon vitamin C treatment (Table 2).

**Table 2:** **Summary of proteins down-regulated in Vitamin C treated cells**.

**Uniprot accession no**.	**Description**	**PLGS score**	Control: Vitamin C Log(e)Ratio^*^
Q3L887	Acyl CoA dehydrogenase fadE5	985.75	Unique^**^
A0R4Z5	Acetyl CoA acetyltransferase fadA6	1177.01	0.97 ± 0.28
A0QXY1	Acyl CoA ligase FadD31	549.12	Unique^**^
A0QU54	Acyl CoA dehydrogenase	619.53	1.35 ± 0.18
A0R2P1	3 Hydroxyacyl CoA dehydrogenase	1710.87	0.95 ± 0.16
A0R618	Acyl CoA synthase fadD32	1844.41	1.02 ± 0.08
A0R4C3	Phosphate ABC transporter phosphate binding protein PstS	3202.45	1.80 ± 0.09
A0R202	ATP synthase alpha atpA	5736.65	0.97 ± 0.06
A0R200	ATP synthase beta atpD	7370.45	1.04 ± 0.06
A0QZ48	Prokaryotic ubiquitin like protein Pup	4303.41	1.10 ± 0.25
A0R006	Cell wall synthesis protein Wag31	5047.6	1.12 ± 0.08
A0R012	Cell division protein FtsZ	2390.89	0.97 ± 0.14
A0R647	Bacterioferritin BfrB	12902.35	1.02 ± 0.06
A0QVZ3	Iron dependent repressor IdeR	4646.21	0.89 ± 0.19
A0QVU2	35 kDa protein	2650.75	1.09 ± 0.13
A0R4D0	Uncharacterized protein	8652.92	1.10 ± 0.1
A0QSK7	Uncharacterized protein	808.68	1.16 ± 0.24

* The natural log of the average relative intensity measurement for associated peptide ions from control divided by the average relative intensity measurement for the same ions found in the Vitamin C treatment.

** Unique indicates a protein not detected in Vitamin C treated cells.

Similarly, Wag31, a homolog of cell shape/cell division protein DivIVA, is downregulated by 3-folds in vitamin C treated cells. It is essential for mycobacterial viability as well as for septal and polar peptidoglycan synthesis and localizes at poles and septa (Kang et al., 2008). Recently, cell division dynamics visualized by using a merodiploid reporter strain expressing a Wag31-GFP (green-fluorescent protein) and time-lapse fluorescence microscopy concluded, cytokinesis and localization of Wag31 to the septum occurs very close together in time (Santi et al., 2013). Wag31 is phosphorylated by Pyruvate kinase, and the phosphorylated form is competent for polymerization and localization to sites of peptidoglycan synthesis (Kang et al., 2005). In our study, along with Wag31, Pyruvate kinase is down-regulated by more than 2.5-fold in vitamin C treated cells (Supporting online Material). The down-regulation of FtsZ and Wag31 also explain the morphological changes like reduction in size observed in ascorbic acid treated *M. smegmatis* cells (Narwadiya et al., 2011).

### Iron related proteins

In an aerobic environment, the two redox forms of iron Fe^+2^ and Fe^+3^ create a dilemma for living organisms. Fe^+2^ activates dioxygen, with the general production of intermediate reactive species causing serious hazards through oxidative damage processes. On the other hand, Fe^+3^ has a low solubility under physiological conditions (~10^−18^ M) requiring living systems to adopt more efficient iron storage/transport/usage mechanisms. Bacterioferritins are ferritins containing haem that performs the role of storage and supply of iron. In our study, down-regulation of bacterioferritin protein BfrB by more than 2.5-fold was observed upon vitamin C treatment. This iron storage protein in *Mycobacterium* sp. has been previously reported to be up-regulated under iron excess condition (Rodriguez et al., 2002) and INH treatment. The decrease in synthesis over degradation ratio of Bfr in presence of vitamin C is as expected, as vitamin C induces dormancy in mycobacterial cells; it makes little sense for the cells to synthesize the iron storage proteins. It can be hypothesized that in presence of vitamin C, cells increase the degradation of bacterioferritin to release stored iron for important functions for example for enzymes involved in respiratory processes. On same line, *IdeR* (Iron dependent regulator) is a dual functional regulator that controls transcription of genes involved in iron acquisition, iron storage and survival in macrophages (Gold et al., 2001). In our study, *IdeR* proteins were down-regulated by 2.5-fold in vitamin C treated cells. Recently, it has been reported that vitamin C pleiotropically affects the biological function and enhances bactericidal activity in the presence of high levels of iron. It seems the degradation of Bfr is implicates the high oxidative environment of the cell which is essential for growth *Mycobacterium* sp (Narwadiya et al., 2011). As described earlier, siderophore molecules are considered as good targets because pathogen survival and virulence is directly related to iron availability.

### Conserved hypothetical

More than 25% of mycobacterial proteome account for conserved hypothetical proteins. These proteins are conserved through *Mycobacterium* sp. but there function is still unknown. Previous studies demonstrates there significant differential expression in different conditions suggest their role in cellular homeostasis but further studies are required to define their role in mycobacteria. In our study, several conserved hypothetical proteins were differentially expressed; an attempt was made to postulate a metabolic role for few of the conserved hypothetical proteins identified based on primary sequence homology using the latest NCBI and Uniprot databases. MSMEG_5830 and MSMEG_2850 were expressed exclusively in vitamin C treated cells. MSMEG_5830, is a homolog of 29 kD antigenic protein, it is also identified as bacteriocin protein in different *Mycobacterium* sp. (like *Mycobacterium neoaurum, Mycobacterium kansasii*, and *Mycobacterium rhodesiae*). MSMEG_2850 is hemerythrin cation binding domain annotated to have cell entry related family protein. MSMEG_2695, MSMEG_5790, MSMEG_1513 are hypothetical proteins which are strongly down regulated in vitamin C treated cells (Table 2). MSMEG_2695 is a 39 kD alanine rich protein which contained a phage shock protein A (PspA) signature domain. Interestingly, PspA from *E. coli* is thought to facilitate the maintenance of the proton motive force and is expressed in response to a variety of environmental stressors, including inhibitors of lipid biosynthesis. Its homolog in *M. tuberculosis*, Rv2744c, was earlier reported to be down-regulated to same extent upon INH treatment. MSMEG_5790 is a homolog of SseC protein known for virulence regulation in *Salmonella* sp. (Klein and Jones, 2001).

## Discussion

Previous reports described that vitamin C is an essential micronutrient which regulates a variety of biological functions. Moreover, it has a strong epidemiological significance in a number of diseases including diseases caused by bacterial pathogens. Mostly effect of vitamin C is ascribed to the host response and very little is known about its direct effect on bacterial physiology. In this study, we attempt to elaborate physiological consequences of vitamin C treatment in *M. smegmatis* by proteome profiling. The complexity of responses seen by proteome profiling is not surprising as a great deal of information is generated and the cells response is not obvious in terms of specific targets. However, this study provides an integrated view of the components of *M. smegmatis* in response to vitamin C. Our dose response studies of vitamin C on mycobacterium concluded that at 2 mM concentration, bacteria attain bacteriostasis. We used 2 mM concentration of vitamin C for further proteome profiling study. In a previous study, 2 mM concentration of vitamin C induced dos-regulon in *M. tuberculosis*, which corresponded to the attainment of threshold dissolved oxygen level in 100 mins. In our study, we found that a major component of dos-regulon, hspX, which is also chaperon protein induced in presence of 2 mM concentration of vitamin C. Furthermore, dormancy regulator Whib3 was also induced at the same concentration of vitamin C. This may be in correlation with induction Phop and Lsr2 which directly regulate expression of Whib3. In our study, we used 2 mM concentration of vitamin C that was 352 μg/ml in solution. Previously various cohort studies debated high dosage of vitamin C from 500 mg- 2 g/day which was found highly effective against heart diseases and strokes.

The key aspects of our study highlights that the metabolic adaptation of *M. smegmatis* upon the treatment of vitamin C was similar to the dormancy metabolism characterized by the previous studies. For example, (1) reversal of TCA cycle, the down-regulation of proteins involved in TCA cycle along with up-regulation of succinate dehydrogenase upon vitamin C treatment as observed earlier in growth arrest implies that the TCA cycle function differed in this condition compare to actively growing cells. The reversal of TCA cycle is an unfavorable and energy consuming process which requires reducing equivalents such as NADH. Noteworthy, levels of NADH is increased in mycobacterial cells during dormancy. Up-regulation of succinate dehydrogenase suggests the irreversible synthesis of fumarate from succinate to maintain redox homeostasis during reductive stress. (2) The re-routing of energy metabolism consequentially decrease the level of ATP in cells along with down-regulation of ATP synthase which is involved in ATP synthesis. The down-regulation of ATP synthesis could be ascribed as a requirement to maintain proton motive force across the cell membrane. This phenomenon is hallmark to the bacilli attaining dormancy. (3) The decrease in iron acquisition and storage proteins is imperative in vitamin C treated bacilli as high concentration of iron in medium will favor pro-oxidant property of vitamin C. At high ferrous concentration vitamin C catalyzes hydroxyl radicals and increase oxidative environment. Overall, induction of important regulators of dormancy and shift in metabolism suggests the proteomic adaptation of bacteria toward the attainment of dormancy phenotype. A limitation of this study includes that it was largely based on abundance of the proteins in particular condition. However, the critical aspect of this study is in line with earlier reports from different groups (Shi et al., 2010; Taneja et al., 2010; Watanabe et al., 2011; Galagan et al., 2013). Moreover, our results are in concordance with mycobacterial response to Wayne hypoxia model and *in vivo* mouse model (Shi et al., 2010; Watanabe et al., 2011; Galagan et al., 2013). Furthermore, it identifies compelling questions regarding redox signaling in mycobacterium which needs critical investigation.

Previous studies provide a foundation to ongoing efforts to include vitamin C to anti-tuberculosis regime (Minnikin et al., 1982; Shi et al., 2010; Abramovitch et al., 2011; Vilchèze et al., 2013). On the other hand, conflicting reports have suggested either beneficial or no effect of vitamin C in the treatment of TB (Stone, 1972). Whether vitamin C is a potent therapeutic or not, is beyond the scope of our study. However, this report highlights the direct effect of vitamin C on bacterial physiology that may serve as a rationale for the further studies in this research area. Additionally, the importance of this study repose in the fact that vitamin C is among the rarest of essential micronutrient which can affect bacterial physiology to this large extent.

## Complementary data

Complete list of differentially expressed proteins in control and vitamin C treated cells.

### Conflict of interest statement

The authors declare that the research was conducted in the absence of any commercial or financial relationships that could be construed as a potential conflict of interest.
